# Recent Advances in the Pathobiology of Hodgkin's Lymphoma: Potential Impact on Diagnostic, Predictive, and Therapeutic Strategies

**DOI:** 10.1155/2011/439456

**Published:** 2011-01-18

**Authors:** Diponkar Banerjee

**Affiliations:** ^1^Centre for Translational and Applied Genomics (CTAG), Department of Pathology, British Columbia Cancer Agency (BCCA), 600 West 10th Avenue, Vancouver, British Columbia, Canada V5Z 4E6; ^2^Department of Pathology and Laboratory Medicine, Faculty of Medicine, University of British Columbia, Vancouver, British Columbia, Canada V6T 2B5

## Abstract

From its first description by Thomas Hodgkin in 1832, Hodgkin's disease, now called Hodgkin's lymphoma, has continued to be a fascinating neoplasm even to this day. In this review, historical aspects, epidemiology, diagnosis, tumor biology, new observations related to host-microenvironment interactions, gene copy number variation, and gene expression profiling in this complex neoplasm are described, with an exploration of chemoresistance mechanisms and potential novel therapies for refractory disease.

## 1. Historical Aspects

In this section a brief overview of the history of the discovery and histologic definition of Hodgkin's lymphoma is presented, highlighting some new observations that shed light on the earlier findings in patients with this disease.

Hodgkin presented seven autopsy cases in his now famous paper “On some morbid appearances of the absorbent glands and spleen” to the Royal Medical and Chirurgical Society of London on January 10 and 24, 1832, the text of which was published in the Transactions of the Medical and Chirurgical Society of London [1]. The full text of his paper has been digitized by Google and is available at http://books.google.ca/ and also at http://www.ncbi.nlm.nih.gov/pmc/articles/PMC2116706/. Although Hodgkin was aware of light microscopy and had used microscopy in an earlier published study with Lister [2], he did not actually examine these cases under the microscope. In 1998, Poston proved by histology and immunohistochemistry that cases II and VI (only cases II, IV, and VI have fixed tissue archived since 1832) indeed represent what we now recognize as Hodgkin's lymphoma (HL) whereas case IV was a case of non-Hodgkin's lymphoma (NHL), possibly a peripheral T cell lymphoma (PTCL) with rare CD15+ Reed-Sternberg-like cells [3]. It is therefore apparent that Thomas Hodgkin had described both forms of lymphoma, and in his paper Poston argues that all lymphomas should be called Hodgkin's lymphoma, subdivided into Reed Sternberg and Non-Reed Sternberg types [3]. The seventh case described by Hodgkin during his presentation was one whose autopsy findings were recorded by Robert Carswell in 1828, elegantly illustrated in watercolor paintings which have been reproduced in Dawson's article “The original illustrations of Hodgkin's disease” [4]. Arguably, Carswell was the first to describe the entity in detail, although Hodgkin makes reference to Malpighi's description of similar lesions in 1666, brought to his attention by a friend, Heming [1].

The first histologic classification of HD was described by Jackson and Parker in 1944 [5]. They divided HD into 3 types, paragranuloma, granuloma, and sarcoma, which are equivalent to nodular lymphocyte predominant, nodular sclerosis, and lymphocyte depleted types, respectively, as recognized in the current classification system (see the following). Modern terminology related to the histopathology of HD was coined in 1966 by Lukes and Butler, who introduced 6 types, including (1) lymphocytic and/or histiocytic (L & H) nodular, (2) L & H diffuse, (3) nodular sclerosis, (4) mixed, (5) diffuse fibrosis, and (6) reticular [6]. In their paper, they reviewed extensively the previous literature on HD and the various terms used by different authors and provide detailed descriptions of Reed-Sternberg cells and their variants. They described the cellular variant of nodular sclerosis and the birefringent bands of collagen in the usual form of NS and proposed that the various histologic types except for NS represent an evolution from L & H nodular to L & H diffuse to mixed, diffuse fibrosis, or reticular forms through a loss of lymphocytes. They correctly interpreted the presence of lymphocytes as evidence of a host response rather than an HD being a mixed lymphoma of small and large cells [6]. This was later reduced to 4 types—lymphocyte predominance (LP), nodular sclerosis (NS), mixed cellularity (MC), and lymphocyte depletion (LD) [7]. 

Current classification systems have replaced the term Hodgkin's disease (HD) with Hodgkin's lymphoma (HL), differentiated between lymphocyte predominant and classical HL, and introduced the subtype lymphocyte rich (LR) classical HL [8, 9].

## 2. Global Incidence, Mortality Rates, and Epidemiology

In this section the patterns of variation in incidence and mortality rates across countries that track and manage HL are discussed.

HL comprises 11% of all lymphomas in the Western world. The estimated incidence of all types of lymphoma in 2010 for Canada was 8,430, of which 7,500 cases are expected to be non-Hodgkin's lymphomas (NHL) and 930 HL [10]. In the United States of America, the estimate for 2009 was 74,490 total lymphomas, including 65,980 NHL, and 8,510 HL [11].

HL had a worldwide incidence of 67,887 cases in 2008, with an age-standardized rate per 100,000 (both genders) of 1.0 [12]. Compared with North America, which has an age-standardized rate (ASR) for HL of 3.2 per 100,000 in whites, HL is relatively rare in Japan (ASR of 0.3 per 100,000 males) and China (ASR of 0.2 per 100,000 males). In developing countries, the incidence of HL is variable and can be higher than in developed countries; for example, the ASR for HL in Yemen is 5.7 per 100,000 males [13].

Considerable regional variation may be seen in several countries. For instance, the highest recorded ASR in the world is in Biella, Italy, at 6.9 per 100,000 males, whereas in Sondrio, Italy, the HL ASR is 1.6 per 100,000 males. Even in low HL ASR countries such as China, regional variation in ASR exists, ranging from a low of 0.1 in Harbin to a high of 0.9 per 100,000 males in Hong Kong [13].

Age-standardized mortality rates are the highest in the Middle East at 1.2 per 100,000 (both genders included) and the lowest in the Western Pacific region at 0.2 per 100,000 (Table 1). The mortality to incidence ratio, a measure of disease severity or effectiveness of therapy, is highest in Africa at 0.9 and lowest in the Americas at 0.2 (Table 1).

**Figure 1:**
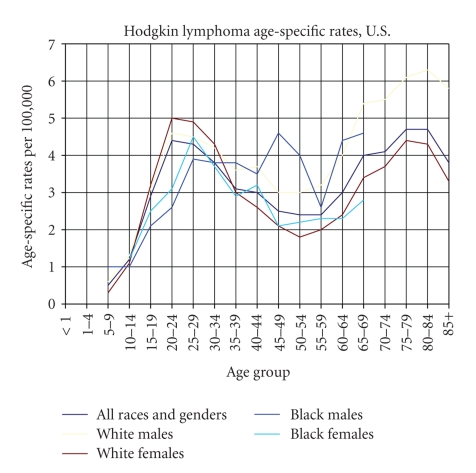
Age-specific rates of incidence (based on data from SEER Cancer Statistics Review) [16].

In developed countries HL is relatively rare in children below the age of 5 years but is the commonest type of cancer in adolescents. It shows a bimodal age distribution with a second peak in patients over the age of 59 years. U.S. Black males seem to show a trimodal age distribution (Figure 1).

The ASR by histological type shows considerable global and regional variation. For instance, the highest ASR for NLPHL is in the Northwest Territories, Canada, at 0.9 per 100,000 males, for LR in Biella Province, Italy, at 0.8 per 100,000 males, for NS also in Biella, Italy at 4.2 per 100,000 males, for MC in Milan, Italy, at 1.4 per 100,000 males, and for LD in Kyadondo County, Uganda, at 0.4 per 100,000 males [13].

The age distribution of histologic subtypes in developed countries is also notable (reviewed in [14]). Childhood cases (defined as ≤14 years of age) of HL tend to be predominantly in males, with MC HL in up to 45% of cases and NLPHL in up to 20% of cases, while in adolescents and young adults (15–35 years of age) and adults (>35 years of age), no gender bias is seen, and the majority of cases (up to 80%) are of the NS histologic subtype. In adults older than 55 years of age, up to 50% are of MC type [14].

The standard histologic and immunohistochemical features of HL have recently been reviewed in detail and illustrated by Eberle et al. [15] and thus not discussed in this paper. It should be noted, however, that there is considerable interobserver variation amongst pathologists regarding the diagnosis and subclassification of Hodgkin's lymphoma, and in the absence of central review by expert pathologists, some of these global statistics may be misleading (see section on interobserver variability). This is further complicated by the fact that there are cases that are not easily classified as they have overlapping features of HL and NHL, so called grey-zone lymphomas (GZL), discussed further in a subsequent section.

## 3. Clinical Presentation

The clinical presentation of HL has changed since its description in the early literature due to the recent developments in the incidence and successful control of HIV+ disease in the Western world. These are discussed in this section. Some of the classic symptoms of alcohol-induced pain may now have a biological explanation. 

A comprehensive discussion of the clinical presentation and natural history of HL has been published by Connors [17]. For the purpose of this review, only the key points are summarized. The clinical presentation of HL depends on the geographic region, age of the patient, and diseases affecting the immune status of the patient. Thus in developed countries, HL presents in adolescents and young adults as localized, persistent, painless, firm lymphadenopathy, most commonly in cervical nodes, supraclavicular nodes, and less commonly in axillary nodes [17]. 

In developing countries, advanced stage and lack of a bimodal age pattern may be seen more often than in developed countries in addition to histologic differences such as a high prevalence of mixed cellularity (MC) HL [18–20]. In immunocompromised patients such as those with human immunodeficiency virus (HIV) infection, HL usually presents as extranodal disease and B symptoms (night sweats, fever, and weight loss), advanced stage, commonly with bone marrow involvement [21]. 

HL is one of the most common non-AIDS defining neoplasms in the HIV+ population and is increasing in prevalence in patients treated with Highly Active Anti-retroviral Therapy (HAART) whereas the incidence of non-Hodgkin's lymphoma has fallen in such patients [22–25]. Older patients above the age of 60 often present with subdiaphragmatic disease, mixed cellularity histology, and B symptoms [17, 26]. 

Rarely, HL may present with paraneoplastic syndromes, including central nervous system, renal, and other manifestations [17]. Historically emphasized paraneoplastic symptoms include pruritus and alcohol-induced pain. Pruritus can occur in up to 30% of patients with HL but is not a specific finding as it can occur in patients with NHL, leukemia, iron deficiency, cutaneous T cell lymphomas, and other malignancies [27–32]. It can be severe and intractable [27, 30, 33, 34]. 

Alcohol-induced pain has been associated with HL since the early 1950s [35–47] but can occur in other conditions such as tuberculous lymphadenitis [48]. Deliberate testing with alcohol was reported to induce pain in 17% of patients with HL [40]. The pain may occur immediately after drinking an alcoholic beverage (the type of alcoholic beverage does not seem to be a factor), or several minutes to hours later, usually, but not always, in involved lymph nodes with radiated pain distribution [40]. It can be severe enough to “reduce the patient to tears” [40]. This symptom is associated with early stage, younger patients, the number of eosinophils, and degree of fibrosis in affected nodes and inversely related to the mitotic count in RS cells [40]. Interestingly, one of the enzymes (aldehyde dehydrogenase) in the alcohol metabolism pathway is implicated in poor outcome in HL patients who have a macrophage gene signature in the tumor microenvironment [49]. Since this enzyme converts acetaldehyde, a product of alcohol dehydrogenase, into acetic acid, it is possible that alcohol-induced pain is due to acetic acid production in the tissues affected by HL heavily infiltrated by macrophages. Acetic acid is commonly used to induce acute visceral pain in mice, a test known as the “acetic acid writhing test”, which, although conjuring images of medieval torture, is used to this day in conscious animals for the evaluation of pain control by analgesics [50].

## 4. Diagnosis

In this section, a brief discussion of the diagnostic approach, pitfalls of cytology, and interobserver variability in histologic assessment are highlighted. The changing definitions and clinical characteristics of grey-zone lymphomas are discussed. Innovations in flow cytometric evaluation of HL may allow better and more rapid diagnosis of HL in the future.

The British Columbia Cancer Agency Cancer Management Guidelines (http://www.bccancer.bc.ca/HPI/CancerManagementGuidelines/Lymphoma/HodgkinsDisease.htm) recommend that the diagnosis be established by open biopsy and not on the basis of fine-needle aspiration biopsy. Hodgkin's lymphoma diagnosis and classification is based on appropriate cellular and tissue architectural findings. Diagnosis may be compromised if the biopsy is insufficient in size or crushed [17]. If there is more than one enlarged lymph node, biopsy of the largest node may be more informative as smaller nodes may only show reactive changes [51].

### 4.1. Role of Fine-Needle Aspiration Cytology

There are numerous reports on the use of fine-needle aspiration (FNA) cytology for the diagnosis of HL [52–73]. Most of these are based on small retrospective series of cases and may suffer from selection bias. FNA diagnosis of HL lesions may be associated with a false negative rate of 15–40% [52, 66, 69]. Generally, because of diagnostic errors that may occur even with the best histologic preparations, FNA samples are not considered reliable enough for the diagnosis and classification of HL. However, this may change as a result of the development of innovative protocols that can identify RS cells by flow cytometry using a 9-color single tube assay.

### 4.2. Diagnostic Pitfalls and Interobserver Variability

There have been a few publications over the years that have dealt with diagnostic errors in pathologic classification of Hodgkin's lymphoma [74–77]. Woodruff reported that 13% of HL cases on review were reclassified as NHL and that 31% of HL cases had been incorrectly subclassified [74]. Miller et al. found that 36 of 287 (13%) cases of HL were actually cases of NHL. Most of the cases erroneously diagnosed as HL were diffuse large cell lymphomas and often extranodal in location [75]. In a recent population-based cancer registry study published, the diagnosis of HL was noted to be incorrect in only 3% of cases reviewed and concluded that cancer registries should not worry too much about diagnostic discrepancies [76]. This study, however, was restricted to female patients in a limited region (Greater Bay area of Northern California) where one would expect that the expertise of regional pathologists might be higher than average in other parts of the world since they had ready and continuous access to R. F. Dorfman, a world-renowned lymphoma pathologist, and a coauthor of that study. Even amongst expert panel members, interobserver variation in the classification of HL can be as high as 8% [78, 79]. In other studies higher error rates were noted when expert reviews were performed [80, 81]. It would appear, therefore, that in countries which do not mandate central review by expert pathologists, the incidence, prevalence, histologic subtypes, and mortality statistics of HL may need correction. Such a central review would uncover cases of grey-zone lymphoma (GZL) as well in a reliable manner.

### 4.3. Grey-Zone Lymphomas (GZLs)

In the latest WHO classification system, a provisional category of B cell neoplasia with features intermediate between diffuse large B cell lymphoma (DLBCL) and cHL was established [9]. GZL seems to be clinically different from standard HL and DLBCL in presentation and response to therapy, although the number of cases reported is quite small so far. Dunleavy et al. presented 14 patients with GZL treated on studies of dose-adjusted infusional etoposide/vincristine/doxorubicin/bolus cyclophosphamide/prednisone plus rituximab (DA-EPOCH-R) [82]. The median age was 30 (range 12–51) years. Seventy one percent were of the male gender, 14% presented with stage III/IV disease (14%), and half the patients had elevated LDH. They described three grey-zone groups, including overlapping features of cHL and primary mediastinal B-cell lymphoma (PMBL) in 9 (64%) patients, cHL and DLBCL in 2 patients (14%), and LPHL and T-cell histiocyte-rich large B cell lymphoma (THRLBCL) in 2 patients (14%). Immunohistochemistry showed the absence of CD10 in almost all cases (only 1 of 14 cases was positive). CD15 was positive in 33–50% and CD30 in 66–100% of cases in the 3 groups. Reed-Sternberg-like cells were seen in GZL with cHL features. Thirteen newly diagnosed GZL patients, as defined previously, received DA-EPOCH-R. Of 11 evaluable patients, 10 (91%) achieved complete response and 1 partial response. At a median follow-up time of 4 years, overall survival and progression-free survival were 86% and 57%, respectively. Of the 9 patients with GZL of the cHL and PMBL overlap type, 4 (44%) also required radiation therapy, a higher percent compared to patients with pure PMBL (3/31; 10%) to achieve durable remissions. They concluded that GZL represents a biological and clinical continuum between HL and B-cell lymphomas and that GZLs are more resistant to treatment than either standard HL or DLBCL and may require more aggressive management including radiation therapy. 

PMBL and HL of NS subtype are now considered related diseases and not included in the current category of GZL [9, 83–89]. It should be also noted that overlap between LPHL and THRLBCL is no longer included as GZL [9], as there is evidence to suggest that the two are also related [90–93]. Lim et al. [91] concluded that THRLBCL is of germinal center B cell derivation in the majority of cases. The lack of t(14;18) and CD10 expression in these cases does not support the notion of histologic progression from a preexisting follicle center cell lymphoma. They found similarities to NLPHL in almost 50% of THRLBCL, but more than half of the cases lacked LP-type neoplastic cells (formerly termed lymphocytic and histiocytic or L & H cells, but now called LP cells) and, instead, contain cells similar to centroblasts or HRS cells, particularly in cases associated with EBV.

### 4.4. Role of Flow Cytometry (FC)

In most clinical laboratories, FC is not routinely used for the diagnosis of HL. This is because RS cells usually make up less than 1% of the lymphoid cells in HL and the microenvironment consists of large numbers of non-neoplastic reactive T and B cells and other cell types. Gating strategies and antibody panels are usually designed for the identification of NHL in tissue biopsies. Fromm et al., however, have reported on the utility of FC in cHL using a 9-color single tube FC assay and successfully sorted RS cells from clinical samples [94, 95]. FC diagnostic sensitivity and specificity were 88.7% and 100%, respectively, when compared to histology [94]. The cases (*n* = 6/53) that were not identified as cHL by FC were due to interpreter error (1 case), sampling error (no RS cells; 2 cases), delayed analysis >72 hours likely leading to loss of RS cells (2 cases), and grey-zone lymphoma ultimately classified on morphology and immunohistochemistry as cHL (1 case). NLPHL is not identifiable by this method [94]. If this can be reproduced by other laboratories, it is conceivable that cHL could be reliably diagnosed in FNA samples in the future when combined with the 9-color assay described by Fromm et al. [94].

## 5. Pathobiology of HL

### 5.1. Cell of Origin

The neoplastic cells of cHL (Hodgkin/Reed-Sternberg cells or H/RS cells) are almost always derived from germinal centre B cells, with rare cases being of T cell origin, while LP HLs (LP cells) are always of germinal centre B cell origin [96–104]. 

Although cHL H/RS cells are derived from germinal centre B cells, they usually lose their B cell programming [105] through a variety of mechanisms including promoter DNA methylation [106–108] and upregulation of *NOTCH1*, a negative regulator of the B cell program [109], whereas non-B cell lineage proteins are upregulated [110]. B cell programming cannot be reconstituted by demethylation of cultured RS cells [111]. The biology of RS cells in cHL has been extensively reviewed recently and thus not discussed in great detail in this review [100, 102, 112].

The LP cell in NLPHL is derived from antigen-activated germinal centre B cells [113]. Like germinal centre B cells, LP cells express functional IgV genes with intraclonal diversification [112], BCL6 protein [114], and GCET1 (centerin), a germinal centre B cell-associated serpin [115]. In contrast to H/RS cells, LP cells generally retain their B cell programming. However, selective loss of the B cell phenotype may also occur in LP cells, including downregulation of CD19, CD37, PAG, and LCK [116–118]. The mechanism is not related to promoter methylation of the encoding genes [118]. Chromosomal breakpoints in IgH and BCL6 associated with a t(3;14)(q27;q32) have been detected in 36% of LP HL [119]. Additional BCL6 rearrangements are frequent in LP HL, associated with t(3;22)(q27;q11), t(3;7)(q27;p12), and t(3;9)(q27;p13) [120].

### 5.2. Role of EBV

The Epstein-Barr virus was first identified by electron microscopy of cultured lymphoblasts from Burkitt's lymphoma in 1964 by Epstein et al. [121] and was identified as the etiologic agent in infectious mononucleosis in 1968 [122, 123].

Weiss et al. showed that 19% of cHL contained the Epstein-Barr (EBV) genome and the infected cells are monoclonal. In situ hybridization confirmed the presence of EBV nucleic acid sequences in RS cells and their variants [124]. Subsequently, several papers confirmed these findings, including the expression of LMP1 by RS cells [125–139]. Up to 40–60% of cHL cases may contain the EBV genome [140].

The fact that EBV is ubiquitous worldwide, infecting 90% of the adult population [141], and is B lymphocytotropic has made it complicated in precisely determining its causative role in HL as it may be a passenger, but not a driver in HL, considering that up to 40%–60% of HL cases do not contain the EBV genome [140]. EBV-associated HD is more common in Hispanics versus whites, mixed cellularity versus nodular sclerosis histologic subtypes, children from economically less-developed versus more-developed regions, and young adult males versus females [142]. 

It is possible that two forms of HL exist, one in immunologically competent patients without association with EBV, and the other in patients with severe or subtle immune dysfunction, where HL is associated with EBV. Even in EBV+ HL, age-related differences in outcome have been described, suggesting that an intact immune system may be beneficial to patients with EBV+ HL. For instance, in young patients (<50 years of age), complete response to therapy and 2-year failure-free survival is significantly better in EBV+ HL cases [143, 144], while in elderly patients, EBV+ cases have a worse outcome [145]. 

Familial HL is associated with EBV positivity and HLA class I haplotype identity [146]. HLA class I region microsatellite markers D6S265 and D6S510 are associated with EBV+ HL while in all HLs including EBV− HL, class III D6S273 marker shows an association [147]. Adverse outcome in EBV− cases is associated with loss of major-histocompatibility-complex (MHC) Class I and class II HLA antigen expression [148]. This suggests that in EBV+ cases, not only is an intact immune system beneficial to patients but also HLA class I polymorphism may influence the persistence of EBV genome, presumably causing variable antigen presentation during the active phase of EBV infection, thus allowing latently infected B cells to survive the initial immune response. EBV− HL cells may survive the immune response by downregulating both class I and class II HLA antigens. HLA-A*02 can present EBV-associated peptides, triggering effective immune responses, and has been shown to have a protective effect (i.e., reduced risk of developing EBV+ HL) [149].

The interaction between EBV+ HL cells and the microenvironment is better understood in the context of EBV biology. EBV gains entry into B cells using the major envelope glycoprotein gp350 which binds to the C3d complement receptor CD21 expressed by B cells [150]. The MHC class II antigen acts as a cofactor for EBV infection of B cells [151]. The binding of viral glycoprotein gp350 to CD21 occurs on the cell surface at which time it is 50 nm from the cell membrane [152]. Internalization involves a complex of three glycoproteins, gH, gL, and gp42. Glycoprotein gp42 binds to HLA-DR [151]. EBV's linear DNA molecule then becomes circular, forming an episome, and persists as a latent infection in memory B cells for years [153, 154].

Whereas 94 viral proteins are encoded by the EBV genome and are expressed during viral replication, most are downregulated when the virus assumes its latent form, so that only 10 continue to be expressed in latent infections [155]. In so doing, the latently infected B cells are less likely to be cleared by the immune response which during the primary infection includes natural killer (NK) cells and cytotoxic T cells [156]. 

The virus deploys additional strategies to escape immune attack, expressing proteins that mimic various receptors and cytokines. For instance, the *BCRF1* gene encodes viral IL-10 (vIL-10), which is highly homologous to human IL-10 and is necessary for B cell transformation by EBV [157]. IL-10 has several properties that promote cell survival, including upregulation of Bcl-2 [158] and inhibition of cytokine production by macrophages and Th1 cells [159, 160]. Certain single nucleotides polymorphisms in the IL-10 gene are associated with worse outcome [161]. This is further discussed in another section hereinafter. 

EBV latent membrane protein LMP1 mimics the CD40 receptor [162] while LMP2a mimics a B cell receptor [163]. For detailed reviews of EBV biology see Cohen [141] and Klein et al. [164]. Comprehensive EBV-human protein interaction maps have been generated by Calderwood et al. [165]. Forty three interactions between EBV proteins and 173 interactions between EBV and human proteins were identified [165], indicating the complexity of interaction between EBV proteins and human proteins, each interaction with possible biologic impact.

### 5.3. Gene Copy Number Variation in HRS Cells

Sub-megabase resolution tiling (SMRT) array-based comparative genomic hybridization profiling revealed gains and losses of 9 novel regions not previously reported in the literature in Hodgkin Lymphoma cell lines L428 and KMH2, which shared gains in chromosome cytobands 2q23.1-q24.2, 7q32.2-q36.3, 9p21.3-p13.3, and 12q13.13-q14.1 and losses in 13q12.13-q12.3 and 18q21.32-q23 [166]. Genes mapping to these regions include cell cycle-associated genes *CDK6*, *PCNA,* and *ATM*, MAPK signaling pathway genes *DDIT3*, *HSPB1,* and *MYC*, genes encoding tight junction proteins *CLDN4* (claudin4) and *PARD6G, *Jak/Stat signaling pathway genes *STAT1*, *JAK2,* and *MYC, *and* ING *tumour suppressor gene *ING3* [166].

Two recent studies looked at gene copy number variation in microdissected HRS cells from clinical samples. Hartman et al. obtained DNA from cHL cases rich in Hodgkin-Reed-Sternberg cells to avoid amplification-associated bias. The cases were either LDHL or NSHL grade II. DNA obtained from about 100,000 microdissected Hodgkin-Reed-Sternberg cells of each of 10 classical Hodgkin's lymphoma cases was hybridized onto commercial (Agilent) 105 K oligonucleotide comparative genomic hybridization microarrays. Gains were identified in at least 50% (5/10 cases) of the cases involving 2p12-16, 5q15-23, 6p22, 8q13, 8q24, 9p21-24, 9q34, 12q13-14, 17q12, 19p13, 19q13, and 20q11. Losses which were recurrent in at least 50% (5/10) of cases were noted affecting Xp21, 6q23-24, and 13q22. Regions of gains include genes such as *STAT6* (12q13), *NOTCH1* (9q34), and *JUNB *(19p13) which are constitutively expressed in cHL [167]. 

The second, more recent, study by Steidl et al. looked at 53 patients, including 23 patients that had failed primary treatment [168]. In this study, fewer RS cells were microdissected from each case (500–1000 cells per case). DNA was extracted and subjected to whole genome amplification (WGA). Array CGH was performed on sub-megabase resolution tiling (SMRT) arrays at a functional resolution of 50 kb. Regions of gain were observed in more than 20% of the cases in 2p, 9p, 12p, 16p, 17p, 17q, 19p, 19q, 20q, and 21q, whereas losses were noted in 1p, 6q, 7q, 8p, 11q, and 13q. Gains of 16p11.2-13.3 were associated with treatment failure and shorter disease-specific survival. One of the genes mapping to this region is the multidrug resistance gene *ABCC1* (encoding multidrug resistance protein MRP1, a member of the superfamily of ATP-binding cassette (ABC) transporters) [169, 170]. In doxorubicin-resistant HL cell line KMH2, Steidl et al. found gains and overexpression of *ABCC1* and siRNA knock-down sensitized KMH2 cells to doxorubicin toxicity *in vitro* [168]. This suggests that primary treatment failure in cHL may be due to amplification and overexpression of *ABCC1*. Confirmation with anti-MRP1 monoclonal antibody immunostaining of primary refractory and treatment sensitive cHL cases might be useful so that this could be used to predict response in cHL by standard immunohistochemistry (IHC).

### 5.4. Gene Expression Studies

In a small number of cases (21) of cHL, using cDNA arrays covering about 1000 genes, Devilard et al. found a signature that can distinguish between good outcome Hodgkin's disease (GOHD) and bad outcome (BOHD) cases [171]. They did not isolate HRS cells by microdissection but used whole cells including the microenvironment. GOHD cases were associated with overexpression of genes involved in apoptotic induction and cell signaling pathways, including cytokines, whereas BOHD was associated with overexpression of genes associated with fibroblast activation, angiogenesis, extracellular matrix remodeling, cell proliferation, and the downregulation of tumor suppressor genes. 

In another study including 29 cases of cHL and over 9000 genes (OncoChip.v2 cDNA microarrays), Sánchez-Aguilera et al. identified 145 genes predictive of outcome [49]. Four different signatures were obtained by supervised hierarchical clustering, 2 of which were associated with the host immune response of tumor microenvironment and the other 2 with the HRS cells based on known expression in HL cell lines and normal germinal centre B cells. 

The immune response signature associated with poor outcome was overexpression of genes expressed by specific subpopulations of T cells such as *CD8B1 *(encoding the CD8 beta chain of cytotoxic T cells, a coreceptor for class I MHC recognition), *CD3D* (encoding the delta subunit of the T cell receptor/CD3 complex), *CD26 *(encoding dipeptidyl-peptidase 4, also known as adenosine deaminase complexing protein-2, or T-cell activation antigen CD26, an intrinsic membrane glycoprotein and a serine exopeptidase), *SH2D1A *(SH2 domain-containing protein 1A, also known as T cell signal transduction molecule SAP, involved in modifying signal transduction pathways in T, B, and NK cells), macrophages such as *ALDH1A1 *(aldehyde dehydrogenase 1 family member A1, an enzyme that is involved in the second step in the alcohol metabolism pathway which converts acetaldehyde to acetic acid), *LYZ *(encodes lysozyme), *STAT1 *(encodes signal transducer and activator of transcription 1), and CD8+ cells such as *ITM2A *(integral membrane protein 2A) which is induced during T cell activation and causes downregulation of CD8 [172]. 

A signature associated with favorable outcome included overexpression of genes relevant to adhesion molecules and remodeling of the extracellular matrix such as *TIMP4* (tissue inhibitor of metalloproteinase 4), *SPON1 *(spondin 1, extracellular matrix protein, which has vascular SMC growth promoting activity [173, 174]), *LAMB1 *(laminin, beta 1; tissue factor (TF) is constitutively associated with laminin-binding beta(1) integrins that support TF-VIIa-PAR2 signaling leading to upregulation of proangiogenic and immune modulatory cytokines and growth factors [175]), genes active in fibroblast function and chemotaxis such as *TACR1 *(tachykinin receptor 1) and *CCL26 *(chemokine (C-C motif) ligand 26; also known as eotaxin-3, one of the chemokines that attract eosinophils into tissues [176, 177]), and molecules expressed by antigen-presenting cells such as *CR1 *(complement component (3b/4b) receptor 1; CD35, expressed on follicular dendritic cells and HRS cells [178]), *HLA-DRB3,* and subpopulations of B cells such as *IRTA2 *(immunoglobulin superfamily receptor translocation-associated protein 2; CD307; an EBNA2-induced Fc-receptor homologue [179]) and *VDR *(VDR agonists can activate CD4(+) CD25(+) regulatory T cells and promote their recruitment to inflammatory sites [180]) [49]. 

Clusters 3 and 4 consisted of genes overexpressed in the unfavorable outcome group and included genes involved in apoptosis regulation such as *CYCS *(cytochrome c, somatic) and *HSPA1L *(heat shock 70 kDa protein 1-like; molecular chaperone that is involved in folding of newly synthesized polypeptides, refolding of misfolded proteins, and translocation of proteins across biological membranes and has regulatory roles in signal transduction, cell cycle, and apoptosis [181]), signal transduction such as *PDCD10* (programmed cell death 10; co-overexpression of PDCD10 can promote cell proliferation and transformation through regulation of the extracellular signal-regulated kinase (ERK) pathway [182]) and *PRKACB *(protein kinase, cAMP-dependent, catalytic, beta), and metabolism and cell growth such as *COX7A2 *(cytochrome c oxidase subunit VIIa polypeptide 2)*, MYCN *(*N-myc*), and* DCK *(*deoxycytidine kinase*), and genes coding for cell-cycle-regulatory proteins such as those involved in the mitotic checkpoint such as *MAD2L1 *(*MAD2 mitotic arrest deficient-like 1*), *BUB1 *(budding uninhibited by benzimidazoles 1 homolog; essential for spindle-assembly checkpoint signaling [183]), *STK6 *(*aurora kinase A*), *CDC2 *(*cyclin-dependent kinase 2*), and *CHEK1 *(*CHK1 checkpoint homolog*). Some of the genes encode enzymes conferring drug resistance such as *RRM2 *(*ribonucleotide reductase M2; *inhibits Wnt signaling [184])*, TOP2A,* and *TYMS* (thymidylate synthetase) [49]. 

In a larger study, Chetaille et al. studied 63 cHL cases (not enriched for HRS by microdissection, thus including cells from the microenvironment) using full transcriptome coverage (Affymetrix U133 A2.0 human oligonucleotide microarrays) and found 47 genes associated with adverse outcome, and 403 genes associated with favorable outcome [185]. Favorable outcome was associated with expressed genes of the “B-cell” cluster, such as *BCL11A* (encoding B-cell CLL/lymphoma 11A, a zinc finger protein), *BANK1* (B-cell scaffold protein with ankyrin repeats), *STAP1* (signal transducing adaptor family member 1), *BLNK* (B-cell linker), *FCER2* (CD23; low affinity II receptor for the Fc fragment of IgE), *CD24*, and *CCL21* (chemokine (C-C motif) ligand 21; chemotactic for T cells), genes of an “apoptosis” cluster such as YWHAB (tyrosine 3-monooxygenase/tryptophan 5-monooxygenase activation protein, beta polypeptide), *CASP8*, *PTPRC* (protein tyrosine phosphatase, receptor type, C), and *TANK* (TRAF family member-associated NFKB activator), and genes of the “cell metabolism” cluster. Genes associated with unfavorable outcome were in the “extracellular matrix” cluster such as collagen genes *COL1A1/4A1/4A2/5A1/18A1*, *THBS1/2* (thrombospondin 1and 2), *FN1* (fibronectin 1), *EDNRA* (endothelin receptor type A), *ITGB5* (integrin, beta 5), and *LAMA4* (laminin, alpha 4) [185].

Global gene expression analysis of microdissected LP cells (L & H cells) from 5 cases of NLPHL has been reported by Brune et al. [186]. The gene expression signatures when examined by principal component analysis (PCA) were closer to T cell-rich B cell lymphoma and classical HL than to diffuse large B cell lymphoma, Burkitt lymphoma, and follicular lymphoma. Forty three genes were differentially expressed in LP cells (using false discovery rate (FDR) filter set at <0.05 after applying the *t* test) in comparison with cHL HRS cells, whereas 295 genes were differentially expressed in comparison with FL cells. By relaxing the FDR filter to <0.1, a total of 129 genes were found to be differentially expressed between LP cells and HRS cells, 123 of which had higher expression in LP cells, and 6 were higher in expression in HRS cells. Of interest is the increased expression of *ABCC1* in LP cells, which, as already discussed, encodes multidrug resistance protein MRP1 [169] and is also amplified and overexpressed in primary treatment refractory cHL [168]. Two other genes of practical relevance include *CTSB* (cathepsin B) and *LGALS3* (lectin, galactoside-binding, soluble), the encoded proteins of which label LP cells in 100% of NLPHL cases [186].

### 5.5. Host Microenvironment/Immune Response

The microenvironment in LP HL is different from that in cHL. An interesting phenomenon has been reported in LP HL, in which a population of CD4+CD8+ CD1a− TdT− (double positive or DP) T cells is detected, comprising up to 38% of T cells in 58% of LP HL cases. Such cells are also found in 38% of cases of progressively transformed germinal centers (PTGCs) but are relatively infrequent (6%) in cHL. Similar DP T cells have been found in melanomas and in breast cancer tissue and pleural effusions associated with breast cancer [187, 188]. They produce TNF-alpha, IL-13, IL-4, and IL-5 at higher levels than conventional mature T cells [187]. Such cells are seen in various other species and are considered to be class II restricted memory CD4+ helper T cells that acquire CD8 alpha chain after activation [189]. Age-dependent increases in DP T cell have been reported in elderly healthy subjects [190].

Although most patients with cHL are cured with first-line therapy, 15%–20% of patients with stage I–II HL and 35%–40% of patients with stage III–IV HL and adverse risk factors relapse after first-line therapy [191, 192]. Patients with relapsed or refractory HL often receive second line, salvage chemotherapy followed by high-dose chemotherapy and autologous stem cell transplant (HDCT/ASCT). Patients who fail HDCT/ASCT have a poor outcome [192–195]. A recent observation of host-response gene signatures highlighted the tumor-associated macrophage gene signature as significantly associated with primary treatment failure. The increased number of CD68+ macrophages identified by IHC was associated with a shortened progression-free survival, an increased risk of relapse after HDCT/ASCT, and shortened disease-specific survival. In multivariate analysis, CD68+ cells as a prognostic factor were superior to the International Prognostic Score for disease-specific survival. The absence of an increased number of CD68+ cells in patients with limited-stage disease predicted long-term disease-specific survival of 100% in cHL patients treated with current treatment protocols [196]. Thus a relatively simply performed IHC assay for CD68, which is widely available in clinical laboratories, can identify patients with HL who are likely to be refractory to first line therapy and is thus the first predictive *in vitro* test for cHL [197]. This also raises the possibility that modulating macrophage numbers and/or function with anti-CD68 or other macrophage-specific monoclonal antibodies might be a therapeutic strategy worth exploring.

### 5.6. MicroRNAs

MicroRNAs are short, noncoding RNAs that are part of RNA silencing pathways that function either by targeting messenger RNAs for degradation or by repressing translation [198]. The *BIC *(B-cell receptor inducible) transcript which contains microRNA-155 (miR-155) is expressed in cHL, activated B cell-like diffuse large B cell lymphoma, and primary mediastinal B cell lymphoma (PMBL) [199]. It has been shown that miR-155 expression is higher in EBV-immortalized B cells than in EBV-negative B cells. LMP1, but not LMP2, upregulates the expression of miR-155 [200]. *AGTR1* (angiotensin II receptor, type 1), *FGF7* (fibroblast growth factor 7), *ZNF537* (TSHZ3; teashirt zinc finger homeobox 3), *ZIC3* (Zic family member 3), and *IKBKE* (inhibitor of kappa light polypeptide gene enhancer in B-cells, kinase epsilon) are miR-155 target genes in cHL [201].

The expression profiling of miRNAs in cHL and reactive nodes revealed 3 groups: nodular sclerosis cHL, mixed cellularity cHL, and reactive lymph nodes (RLNs). Twenty five miRNAs differentiated cHL from RLNs, and 36 miRNAs were differentially expressed in the nodular sclerosis and mixed cellularity subtypes. MiR-96, miR-128a, and miR-128b were selectively downregulated in cHL containing the EBV genome [202]. 

*PRDM1/blimp-1* (PR domain containing 1, with ZNF domain; Beta-interferon gene positive regulatory domain I-binding factor) is a transcriptional repressor that binds specifically to the PRDI element in the promoter of the beta-interferon gene and drives the maturation of B-lymphocytes into immunoglobulin secreting cells. It is a target for downregulation by miR-9 and let-7a in HRS cells of cHL. MiRNA-mediated downregulation of *PRDM1/Blimp-1* may contribute to the loss of the B cell program in HRS cells by interfering with normal B-cell terminal differentiation [203].

Patients with cHL demonstrating low miR-135a expression have a higher probability of relapse and a shorter disease-free survival. Downregulation by miR-135a of JAK2, a cytoplasmic tyrosine kinase involved in cytokine receptor signaling pathways, causes decreased mRNA and protein levels of the antiapoptotic gene *Bcl-xL*, supporting a possible role for *Bcl-xL* in miR-135a/JAK2-mediated apoptosis [204].

Microdissected HRS cells, when compared to CD77+ germinal centre B cells, assessed for a panel of 360 miRNAs, show upregulation of 12 miRNAs including miR-155 (targets *AGTR1, SOCS1, CD47, NAMPT, IKBKE, BCL11A*), miR-21 (targets *PTEN, BCL11A, BCL2, CD47*), miR-20a (targets *TGFBR2, SMAD5, SOCS6*), miR-9 (targets *BCL11A, BCL6, NFKB1, CD47, SOCS5*), and miR-16 (targets *BCL2, CD164, SOCS6*) and downregulation of 3 miRNAs miR-614 (targets *FOXD1*), miR-200a# (targets* ZEB1, ZEB2, STAT4, STAT5A, STAT5B, JUN*), and miR-520a# (targets *BCL6, BTG1, PBX3, BCL11A, BCL11B*) [205]. These are interesting observations, since several targets of upregulated miRNAs are themselves regulators of lymphocyte function. For instance, SOCS1, SOCS5, and SOCS6 are members of the suppressors of cytokine signaling family [206], and thus silencing by miRNAs should lead to increased cytokine signaling and response due to reduction in suppression of cytokine signaling. *PTEN* silencing may lead to unopposed phosphoinositide 3-kinase (PI3K) activity which may contribute to the development of lymphomas [207].

### 5.7. Single Nucleotide Polymorphisms (SNPs) in Selected Genes in HL

Nieters et al. have looked at SNPs in genes encoding the family of Toll-like receptors (TLRs), IL10 and IL10RA in 710 patients with various forms of lymphoma, and matched controls and found that the IL10RA Ser138Gly variant had a 50% protective effect against Hodgkin's lymphoma (HL) [208]. Hohaus et al. looked at IL10 plasma levels and SNPs in the IL10 gene promoter in patients with HL and found that patients with high IL-10 levels were tended to have advanced stage and worse outcome. Variant alleles at position-592 (AA) and -1082 (GG) of the IL-10 promoter were associated with increased IL-10 plasma levels [209]. In an earlier study, Hohaus et al. had found by multivariate analysis that the IL-10-592AA and the IL-6-174GG genotypes were independent prognostic factors in HL patients [161].

Chang et al. have reported that regular use of aspirin reduces the risk of developing HL [210], and since aspirin inhibits the activation of the transcription factor nuclear factor-*κ*B (NF-*κ*B) via inhibition of I*κ*B kinase *β*, an activator of NF-*κ*B [211], they investigated whether SNPs in genes involved in nuclear factor-*κ*B (NF-*κ*B) activation and inhibition, other inflammatory pathways, and aspirin metabolism influence HL risk in 473 patients with cHL and 373 controls [212]. They found that HL risk was significantly associated with refSNP ID rs1585215 in *NFKB1* (AG versus AA and GG versus AA) but could not find an association with aspirin use or with SNPs in aspirin-modulated genes *IKKA/CHUK, PTGS2/COX2, CYP2C9*, *UGT1A6*, or* LTC4S *[212]. In a subsequent case-controlled study of Danish patients with HL, they found a protective effect of low-dose aspirin, but not other nonsteroidal anti-inflammatory drugs NSAIDs, against Hodgkin lymphoma development [213].

Polymorphisms in 3 nucleotide excision repair pathway genes (*XPD* [Lys751Gln], *XPC* [Lys939Gln], and *XPG* [Asp1104His]), the base excision repair *XRCC1* (Arg399Gln), and double-strand break repair *XRCC3* (Thr241Met) were studied in 200 HL patients and 220 matched controls. A positive association was found between combined *XRCC1/XRCC3* and *XRCC1/XPC* polymorphisms and risk of HL [214].

### 5.8. Mechanisms of Chemoresistance

As previously discussed, both LP and HRS cells in clinical samples overexpress the multidrug resistance gene *ABCC1*, and HRS cells contain increased copy number of the *ABCC1* gene [168, 186]. Shafer et al. have shown that primary HL tumor samples contain a population of cells (0.2% to 6.5%) with the ability to increase efflux of Hoechst 33342 dye and are resistant to gemcitabine, a commonly used drug for the treatment of refractory HL. These cells not only have the phenotype of HRS cells but also express multidrug resistance genes *ABCG2* and *MDR1 *(*ABCB1*) [215]. Thus HRS cells may be using up to 3 different multidrug resistance proteins to survive the effects of chemotherapy.

Staege et al. have studied the sensitivity of HL cells for cytotoxic drugs (cisplatin, etoposide, melphalan) and compared the gene-expression profiles of chemoresistant and sensitive cell lines. Genes upregulated in resistant cells include those encoding cytokine receptors (*IL5RA*, *IL13RA1*), markers expressed on antigen-presenting cells (*CD40*, *CD80*), as well as genes with known association to chemoresistance, such as myristoylated alanine-rich protein kinase C substrate and *PRAME* (preferentially expressed antigen in melanoma) [216].

Kashkar et al. have reported that chemoresistance in HL is related to XIAP (X-linked inhibitor of apoptosis) as an NF-kappaB-independent target of bortezomib. Low-dose bortezomib sensitizes HL cells against a variety of cytotoxic drugs without altering NF-kappaB activity [217].

## 6. Novel Therapies and New Targets for Drug Development

As the prognosis of HL patients who relapse after HDCT/ASCT is poor, several novel therapies have been or are being assessed for chemoresistant patients, including anti-CD20 and anti-CD30 antibodies, lenalidomide, histone deacetylase inhibitors, mammalian target of rapamycin (mTOR) inhibitors, cytotoxic T-lymphocyte therapy, and reduced-intensity allogeneic transplants (reviewed in [218]). Several of these approaches are highlighted in this section.

### 6.1. Rituximab

Since LP cells in NLPHL express CD20, the role of the anti-CD20 monoclonal antibody, rituximab (Rituxan), has been explored in NLPHL in the past few years, starting with a few cases studies such as that reported by Čulić et al. [219] in which a pediatric patient refractory to ABVD plus radiation therapy achieved complete remission following rituximab infusion. Zojer et al. reported an adult patient with NLPHL who responded to another anti-CD20 antibody, yttrium-90-labelled ibritumomab tiuxetan (Zevalin) [220]. Schulz et al. have reported the results of the German Hodgkin Study Group (GHSG) phase II trial investigating the role of rituximab on refractory or relapsed patients with NLPHL. The overall response rate was 94% in 15 patients confirmed to have NLPHL after central pathology review, including a 53% complete response rate [221]. In another trial, from Stanford University, including treatment-naive patients as well as refractory or relapsed patients, Ekstrand et al. found an overall response rate of 100% and a complete response rate of 41%, but the duration of response was short (median time to progression 10.2 months) [222]. NLPHL successfully treated with rituximab may be associated with relapse with CD20 negative T cell rich B cell lymphoma [223], and thus the long-term outcome of rituximab-treated patients with NLPHL remains to be determined.

Rituximab therapy has also been investigated for the treatment of cHL. This may seem odd since HRS cells do not generally express CD20 due to loss of the B cell program [224–226], but the rationale is based on the hypothesis that a B cell rich tumor microenvironment is associated with favorable outcome [185, 196]. This does not make sense because, if small B cells in the microenvironment are responsible for better outcomes in HL, then destroying them with anti-CD20 antibodies would be expected to be detrimental to outcome. Others have argued that small B cells in the microenvironment deliver survival signals to HRS cells including ligands for CD30 and CD40 and suppress T cell-mediated immune responses by producing IL-10 (reviewed by Oki and Younes [227]) and that clonotypic circulating B cells may be precursors to HRS cells [228]. Pilot studies have demonstrated positive initial results when rituximab was used as a single agent for relapsed or refractory disease [229]. One could argue that this is conceivably due to shrinkage of affected nodes just due to elimination of normal B cells which vastly exceed the number of HRS cells in affected nodes, and not a true response by HRS cells. Clinical trials, however, have shown some promise. In a phase II study of relapsed or refractory cHL the activity of gemcitabine and rituximab was investigated in 33 patients, with objective responses in 48% of the patients, but the median duration of failure-free survival was only 2.7 months [230]. Rituximab plus gemcitabine, ifosfamide, and oxaliplatin (R-GIFOX) has been reported as a less toxic, cytoreductive, and stem cell mobilizing alternative to cisplatin-based salvage regimes, useful not only for pre-ABMT cytoreduction but also for a full salvage treatment program to be safely delivered to patients unfit for high-dose procedures [231]. Rituxan plus ABVD is associated with an event-free survival significantly better than ABVD without rituxan in both patients with IPS scores of 0–2 and IPS scores >2 [232, 233]. A new GHSG trial (HD18) enrolling patients will be evaluating individualization of treatment according to early response to chemotherapy. Patients with IIB (with large mediastinal mass or extranodal infiltration), III A/B, and IV A/B HL that show PET-positive lesions after two cycles of chemotherapy will receive eight cycles of escalated BEACOPP + rituximab (BEACOPPesc + R) and will be compared to those receiving eight cycles of chemotherapy (BEACOPPesc) alone with the primary endpoint being progression-free survival (PFS). For patients with PET-negative lesions after two cycles of chemotherapy the experimental arm (four cycles of BEACOPPesc) will be compared against standard treatment (eight cycles of BEACOPPesc) with the primary endpoint of PFS.

### 6.2. Anti-CD30 Monoclonal Antibodies

A variety of early anti-CD30 preparations have been tried with little or at the most modest effects [218]. Toxicities are significant, including grade 3 to 5 pneumonitis, grade 1 to 2 fatigue, neutropenia, peripheral neuropathy, nausea, diarrhea, and pyrexia, febrile neutropenia, grade 3 vomiting, and grade 4 hyperglycemia, and severe hematologic toxicities (with radioimmunoconjugates) [218]. However, currently, a number of new preparations such as SGN-30, SGN-35, and MDX-060 have been or are being evaluated.

### 6.3. SGN-30

SGN-30 is a chimeric monoclonal antibody with humanized heavy and light chain constant domains. It initially showed activity against HL cell lines in vitro and in xenograft models [234] and was also shown to be able to sensitize HL cell lines to chemotherapeutic agents [235]. The in vivo effects of SGN-30 seem to be dependent on Fc-receptor positive macrophages [236]. In a phase II study on monotherapy with SGN-30 in refractory or relapsed patients, however, objective responses were not observed in HL patients, although a few patients (29%) had stable disease (duration 62–242 days). Toxicity was common but mild or moderate [237].

### 6.4. MDX-060

MDX-060 is a fully human IgG_1_ kappa monoclonal antibody reactive against CD30 [238] and, although well tolerated, has modest activity (6% response) in patients with HL [239].

### 6.5. SGN-35

SGN-35 (brentuximab vedotin) is an antibody-drug conjugate (ADC) containing the potent tubulin inhibitor, monomethylauristatin E (MMAE), linked to the anti-CD30 chimeric monoclonal antibody, cAC10 [240]. MMAE is a derivative of the cytotoxic tubulin modifier auristatin E and was covalently coupled to cAC10 through a valine-citrulline peptide linker. The ADC is stable as there is only a 2% release of MMAE following prolonged incubation in human plasma. However, after receptor-mediated internalization, it is cleaved by lysosomal proteases, which leads to the release of MMAE into the cytosol. It then induces G2/M-phase growth arrest and cell death through the triggering of apoptosis [240]. In a phase I trial 29 patients including 26 with heavily treated relapsed or refractory HL were treated with increasing doses of SGN-35. Outpatient infusions of SGN-35 were generally well tolerated. Of the 28 evaluable patients clinical benefit (PR + SD) was observed in 20 patients (71%) [241]. In a subsequent report, of 28 evaluable patients treated at doses ≥1.2 mg/kg, 46% (*n* = 13) were noted to have an objective response (CR + PR), with a CR rate of 25% (*n* = 7). Two additional PRs were observed in the cohort that received a lower dose (0.6 mg/kg). Median response duration at the time of the report was 22 weeks [242]. A recent press release from Seattle Genetics Inc. and the Takeda Oncology Company indicates that in a pivotal trial of single-agent brentuximab vedotin (SGN-35) conducted in 102 relapsed or refractory HL patients, 75 percent of patients achieved an objective response (not further broken down into CR, PR, SD rates), with a median duration of response greater than six months (http://www.news-medical.net/news/20100927/Takeda-announces-SGN-35-ADC-trial-results-in-Hodgkin-lymphoma-patients.aspx). Results from the pivotal trial are expected to be presented at the 2010 ASH annual meeting. A current trial is also evaluating the combination of SGN-35 and ABVD (http://www.lymphomainfo.net/news/studies-and-trials/seattle-genetics-begins-combination-trial-of-sgn-35-plus-abvd). These trials appear to provide some options for heavily treated HL patients who have failed current therapies including HDCT/ASCT. A recent publication showed that MMAE is released from SGN-35 within CD30+ cancer cell lines and is able to exert cytotoxic activity on bystander CD30− cell lines due to its ability to cross plasma membranes [243]. This might explain the superior clinical responses seen with SGN-35 compared to unconjugated anti-CD30 antibodies. However, theoretically, this might limit the dose of SGN-35 used clinically as it is unclear whether the released MMAE can have systemic nonspecific cell damage and resulting toxicity.

### 6.6. Bendamustine

Bendamustine is a mechlorethamine derivative with a purine-like benzimidazole ring and has structural similarities to alkylating agents and antimetabolites but is non-cross-resistant with other alkylating agents and other drugs [244, 245]. Although it regained importance through its utility in treating rituximab resistant indolent B cell NHL [246], recently it has been reported that bendamustine is highly active in heavily pretreated refractory or relapsed HL and enables referral to nonmyeloablative allogeneic stem cell transplant (NMT) in a significant number of eligible patients [247].

### 6.7. Revlimid

Preliminary findings have been presented on Phase II study including 35 evaluable patients with refractory or relapsed HL treated with single agent lenalidomide (Revlimid), an immunomodulatory drug. The authors observed 1 CR, 5 PR, and 6 SD greater than 6 months. The overall response rate was 17% and the overall cytostatic response rate was 34%. Median duration of CR/PR was 4.5 (range 2–10) months [248]. In another report single-agent lenalidomide was well tolerated and effective in 12 patients with refractory or multiple relapsed HL. All patients showed disease stabilization, and 50% responded (CR + PR) to, including one patient with complete remission even after 24 months at the time of the report. An international phase II study of single agent lenalidomide in relapsed HL patients is being planned, and an international phase I/II study of lenalidomide combined with conventional chemotherapy (AVD-Rev) for elderly HL patients has begun [249].

### 6.8. HDAC Inhibitors

As noted in a previous section, epigenetic changes are responsible for the silencing several B lineage genes in HRS cells, likely helping these cells to survive usual apoptotic pressures and to avoid immune-mediated destruction [106–108, 250]. With this in mind, several investigators have looked at the effects of histone deacetylase (HDAC) inhibition on HRS in vitro, and these studies have led to clinical trials of HDAC inhibitors [251–254]. A study of the expression of HDAC isoforms in HL tissue microarrays has shown that HDAC isoforms 1, 2, and 3 are highly expressed in HL, and reduced HDAC1 expression is associated with inferior outcome in HL [255]. MGCD0103 is an oral isotype-selective inhibitor of histone deacetylases (HDACS) with activity in preclinical models of hematopoietic cancers and has been studied in a phase II trial in patients with relapsed/refractory classical HL, resulting in a 45% overall response [256].

### 6.9. CYB5B

A recently reported 21 kDa protein [257], identified as CYB5B [258], which is normally expressed in the outer mitochondrial membrane, is overexpressed in cHL in the cytoplasm and plasma membrane of HRS cells but not at the plasma membrane of normal reactive lymphocytes or bone marrow precursor cells [258]. Array CGH using a submegabase resolution tiling array revealed gains in the *CYB5B* locus in HL cell lines KMH2 and L428 [258]. HL cell lines show increased CYB5B mRNA but reactive lymphocytes and bone marrow precursor cells show no increase in CYB5B mRNA [258]. By its location at the plasma membrane only in neoplastic cells in cHL, diffuse large B cell lymphoma (DLBCL), and anaplastic large cell lymphoma (ALCL), CYB5B might be an attractive target for antibody-based therapy as toxicity should be minimal since normal, reactive lymphocytes and CD34+ bone marrow precursor cells do not express the protein at the plasma membrane [258].

## 7. Conclusions

One hundred seventy eight years after its discovery by Thomas Hodgkin, Hodgkin's lymphoma continues to fascinate us. Although derived from germinal centre B cells, the neoplastic cells of HL appear to have rid themselves of the normal B cell program and avoid what should normally lead to triggering of programmed cell death through a seemingly symbiotic relationship with its microenvironment which is unlike other types of B cell lymphomas. The mechanisms of chemosensitivity and chemoresistance are beginning to be understood but managing patients who have primary chemorefractory disease or relapse after the most aggressive salvage therapies continues to be a challenge. Novel therapies are being investigated in order to achieve superior outcomes in chemorefractory patients.

## Figures and Tables

**Table 1:** Age-standardized incidence and mortality rates ranked by mortality to incidence ratio (case fatality ratio). Calculations are based on data from GLOBOCAN 2008 [12].

Region	Numbers per year	Incidence ASR	Deaths per year	Mortality ASR	Mortality to incidence ratio
WHO Africa region	5879	0.9	4893	0.8	0.9
WHO East Mediterranean region	7663	1.4	6004	1.2	0.9
Less developed regions	40137	0.7	23698	0.5	0.7
WHO South-East Asia region	11682	0.7	6276	0.4	0.6
WHO Western Pacific region	8476	0.4	3478	0.2	0.5
World	67887	1	30205	0.4	0.4
WHO Europe region	19342	2	5898	0.5	0.3
More developed regions	27750	2	6507	0.4	0.2
WHO Americas region	14802	1.5	3649	0.3	0.2
